# HIV Incidence and Associated Risk Factors Among Young Men Who Have Sex With Men in Tianjin, China: Retrospective Cohort Study

**DOI:** 10.2196/66487

**Published:** 2025-03-19

**Authors:** Yan Guo, Hui Gong, Xiangyu Yan, Xinying Zhang, Tielin Ning, Zhongquan Liu, Long Li, Jie Yang, Liu Li, Changxu Ma, Zhuang Cui, Maohe Yu

**Affiliations:** 1 Tianjin Centers for Disease Control and Prevention, 6 Hua Yue Road, Hedong District, Tianjin, 300011, China, 86 13821181011; 2 School of Disaster and Emergency Medicine, Tianjin University, Tianjin, China; 3 Tianjin Hongqiao Center for Disease Control and Prevention, Tianjin, China; 4 Shenlan Public Health Consulting Service Center, Tianjin, China; 5 Department of Epidemiology and Biostatistics, Tianjin Medical University, Tianjin, China

**Keywords:** young men who have sex with men, YMSM, incidence, HIV, cohort study, risk factor

## Abstract

**Background:**

Young men who have sex with men (YMSM) have a higher risk of HIV infection. However, evidence of HIV incidence from large cohort studies is limited in this key population, particularly among Chinese YMSM.

**Objective:**

This study aimed to investigate the HIV incidence and associated risk factors among YMSM aged 16‐24 years in Tianjin, China.

**Methods:**

We conducted a retrospective cohort study among men who have sex with men aged 16‐24 years from October 2017 to December 2022 through the largest local nongovernmental organization serving men who have sex with men in Tianjin. Participants who responded to the investigations at least twice during the study period were included. They completed questionnaires on demographic information, sexual behaviors, psychoactive substance use, and sexually transmitted infection status. Simultaneously, their blood samples were collected for HIV testing. HIV incidence was calculated by dividing the sum of observed HIV seroconversions by the observed person-years (PYs). A Cox proportional hazards regression model was used to identify risk factors associated with HIV incidence.

**Results:**

A total of 1367 HIV-negative YMSM were included in the cohort, among whom 62 seroconversions occurred, contributing 2384.2 observed PYs; the total incidence was 2.6 (95% CI 2.0‐3.2) per 100 PYs. The segmented HIV incidence rates were 3 (95% CI 1.5‐4.5), 2.4 (95% CI 1.5‐3.3), and 2.7 (95% CI 1.5‐3.9) per 100 PYs for 2017‐2018, 2019‐2020, and 2021‐2022, respectively. Compared to YMSM who had been followed up fewer than three times, those followed up three or more times had a relatively lower risk of HIV infection (Adjusted hazard ratio [AHR] 0.553, 95% CI 0.325‐0.941). YMSM who preferred finding sexual partners offline had a higher risk of HIV infection compared to those who preferred finding sexual partners online (AHR 2.207, 95% CI 1.198‐4.066). Compared to YMSM without syphilis, those infected with syphilis had an increased risk of HIV infection (AHR 2.234, 95% CI 1.137‐4.391). Additionally, YMSM who used psychoactive substances had a higher risk of HIV infection compared to those who did not use such substances (AHR 2.467, 95% CI 1.408‐4.321).

**Conclusions:**

Our study complements data on HIV incidence among YMSM in large cities across China. Syphilis infection and the use of psychoactive substances were risk factors associated with HIV occurrence, demonstrating an urgent need for tailored prevention and control interventions for this key population.

## Introduction

The Global AIDS report released by the Joint United Nations Programme on HIV/AIDS (UNAIDS) [1] estimated that there were 350,000 new HIV infections among people aged 15‐24 years in 2022. Young people accounted for 26% of HIV infections in the Asia-Pacific region in 2020; among them, 52% were men who have sex with men (MSM), making them a key population that requires focused attention for HIV prevention. Meanwhile, the prevalence of HIV among young men who have sex with men (YMSM) has more than doubled in Indonesia (ie, from 6% in 2011 to 13% in 2019) and nearly tripled in Malaysia (ie, 6% in 2012 to 15% in 2022), and Vietnam (ie, 3% in 2011 to 11% in 2020) [2]. Previous studies suggest that young people from key populations experience high vulnerability and challenges in staying healthy and safe in situations marked by stigma, discrimination and harassment, punitive laws, and social taboos [2]. Moreover, young people living with HIV have the lowest rate of awareness about their infection status [3].

Meanwhile, in recent years, the HIV epidemic in China has risen rapidly among young people. The number of new HIV cases reported yearly among people aged 15‐24 years increased from 9373 in 2010 to 15,790 in 2019; a total of more than 140,000 HIV cases have been reported in this age group [4]. HIV has become a major infectious disease that endangers the health of young people in China, particularly in some regions where the proportions of reported cases have increased [4]. The spread of HIV among young people is concerning, posing a serious threat to their lives and health. Due to them being in a stage of rapid physical and mental development, lack of sexual health knowledge, and the increase in high-risk behaviors, they are more vulnerable to HIV.

Given that YMSM are susceptible to HIV infection [5,6], studies have shown that HIV infection rates among YMSM are higher than those in older populations [5-8]. YMSM under 25 years of age represent a demographic with increasing numbers of new HIV infections even as HIV incidence is declining globally [9]. Although there were some data on HIV prevalence among YMSM [9,10], the epidemic remains poorly defined among in this population [11], particularly the data of incidence is scarcely reported. Understanding HIV incidence—especially among subgroups of key populations—is essential for effectively targeting strategies to prevent new HIV infections [12].

Tianjin, located in the Bohai Gulf, is the second most populous city in northern China and one of the four major municipalities, with 16 districts and a resident population of 13.73 million as of 2021 [13,14]. The majority of young HIV cases in Tianjin were attributed to homosexual transmission, and the prevalence of HIV and syphilis among YMSM in the city is high [15]. However, the incidence of HIV among YMSM in Tianjin remains unknown.

This study aimed to (1) identify the HIV-related behavioral characteristics of YMSM, (2) understand the trends of HIV incidence among YMSM, and (3) investigate the association between related factors and HIV seroconversion among YMSM. The findings of this study will provide policymakers with evidence to inform prevention strategies aimed at controlling the HIV epidemic among YMSM.

## Methods

### Study Design

A retrospective cohort study on HIV incidence among YMSM was conducted in Tianjin from 2017 to 2022. First, to enhance geographical coverage and participant accessibility, seven voluntary HIV counselling and testing sites were set up for on-site surveys. Second, the on-site surveys were conducted at Shenlan, the largest local nongovernmental organization (NGO) serving MSM. The surveys were implemented by professional staff at the NGO, who also comprised MSM. To ensure the quality of the surveys, all professional staff at the 7 voluntary HIV counselling and testing sites received systematic and standard training; furthermore, the surveys were carried out through a unified method. Third, to guarantee the representativeness of the participants, two recruitment approaches were adopted. One approach involved professional staff recruiting YMSM through various strategies, such as offline hotspots (eg, gay baths, gay bars, public toilets) and online platforms (eg, WeChat, QQ, Blued). The second approach involved mobilizing the YMSM recruited through the first approach to introduce new YMSM. Fourth, all participants were required to participate in a baseline survey, followed by a follow-up survey every 6 months.

During the study, the fingerprints of the participants’ right index fingers were collected, and a unique fingerprint code was assigned. A verification code was sent to the participants’ cell phones to verify their phone numbers. After successful verification, training investigators conducted face-to-face questionnaire interviews. After the completion of the questionnaires, professional blood collectors obtained venous blood samples from the participants. The blood was sent to a professional laboratory for HIV testing. HIV test and questionnaire responses were linked using the participant’s fingerprint code, which was unique and served as a proxy identifier to match the HIV testing results to each participant. Participants’ fingerprint codes were recorded into an unidentifiable ID number to make the dataset anonymous. The Tianjin Centers for Disease Control and Prevention questionnaire was developed for the target population and presurvey.

### Participant’s Eligibility Criteria

The target population of this study were YMSM who participated in the survey and resided in Tianjin during the study period. Eligible criteria for study enrollment included: (1) born a male biologically, (2) aged 16‐24 years old, (3) who engaged in penetrative oral or anal sexual intercourse with males in the past year, (4) voluntarily consented to questionnaires, fingerprint registration, blood sample collection, and related testing, and (5) self-reported negative HIV tests or were unaware of their HIV infection status before participation in the survey.

### Questionnaire Interview

The questionnaire included 4 parts. The first part collected information on sociodemographic characteristics such as age, residential district, occupation, education, marital status, and duration of residence. The second part collected information on sexual orientation and sexual behaviors in the last 6 months, including heterosexual behaviors, homosexual behaviors, commercial homosexual partners, primary venue to find sexual partners, and condom use. The third part collected information on the use of psychoactive substances (eg, Rush and Capsule Zero), injection drug use in the past year, syphilis infection status, and peer education received in the past year. As smoking, alcohol consumption, and psychoactive substance use are addictive in nature, and previous research by Guo et al [16] showed a positive relationship between smoking and psychoactive substance use among YMSM, this study further explored these relationships. In this study, drinking was defined as consuming alcohol at least twice within the past 30 days, while smoking was defined as continuous or cumulative smoking for at least 6 months before the survey and smoking within the past 30 days.

### HIV Laboratory Test Procedure

Venous blood samples were collected from the study participants. First, rapid HIV test reagents were used; those who tested reactive were retested using ELISA (enzyme-linked immunosorbent assay). If reactive, they were subjected to an HIV supplemental test. The supplemental test was first confirmed using the Western Blot method. If the Western Blot yielded indeterminate or negative results in the confirmatory test, the participants underwent a quantitative nucleic acid test using viral load detection reagents. Participants with nonreactive findings in the initial rapid test or the enzyme immunoassay were further tested using pooled nucleic acid tests.

### Statistical Analysis

Based on the baseline data, frequency analyses stratified by HIV seroconversion status were performed. χ^2 ^tests were used to evaluate the differences in characteristics between participants with and without HIV seroconversion. For HIV seroconversions, the midpoint between the last negative and the first positive test dates was assumed to be the estimated onset date of HIV infection. For participants who did not seroconvert (ie, test positive for HIV), the date of their last survey was used as the endpoint for our analysis. Individual observation time was calculated as the interval between the participant’s baseline survey and their latest HIV test during the study period. HIV incidence was calculated by dividing the number of observed HIV seroconversions by the total observed person-time. The 95% CI for HIV incidence were calculated using person-years (PYs) over the observed time as the denominator. The proportional hazards assumptions for the Cox models were tested by examining the interactions between variables included in the model and the logarithm of the follow-up time; no violations were observed. Therefore, Cox proportional hazards models were used to estimate the associations between various factors and HIV incidence. A multivariate analysis was conducted based on variables identified in univariate analysis; marginally significant variables (*P*≤.10) in univariate analysis and factors that may be associated with new HIV infections were included in a multivariate Cox proportional hazards regression model to identify factors associated with HIV incidence. All analyses were performed using SPSS software (version 24.0; IBM Corp). A two-tailed *P*<.05 was considered statistically significant.

### Ethical Considerations

This study was approved by the Ethics Committee of Tianjin Centers for Disease Control and Prevention (approval number: TJCDC-R-2023‐020) and was carried out in accordance with the Declaration of Helsinki. Ethical guidelines were followed during all the stages of this study. Comprehensive details about the study were provided to the participants during their first survey, and participation in the study was entirely voluntary and all participants provided written informed consent. Participants had the right to opt out of the study at any time. When accessing the dataset of this study, it was entirely deidentifed, and there was no effective way to link the data to the participants; further, the data were completely anonymized during data processing and statistical analyses. We did not pay any monetary compensation to the participants; however, we provided free HIV counseling and testing services.

## Results

### The Cohort

**Figure 1.**
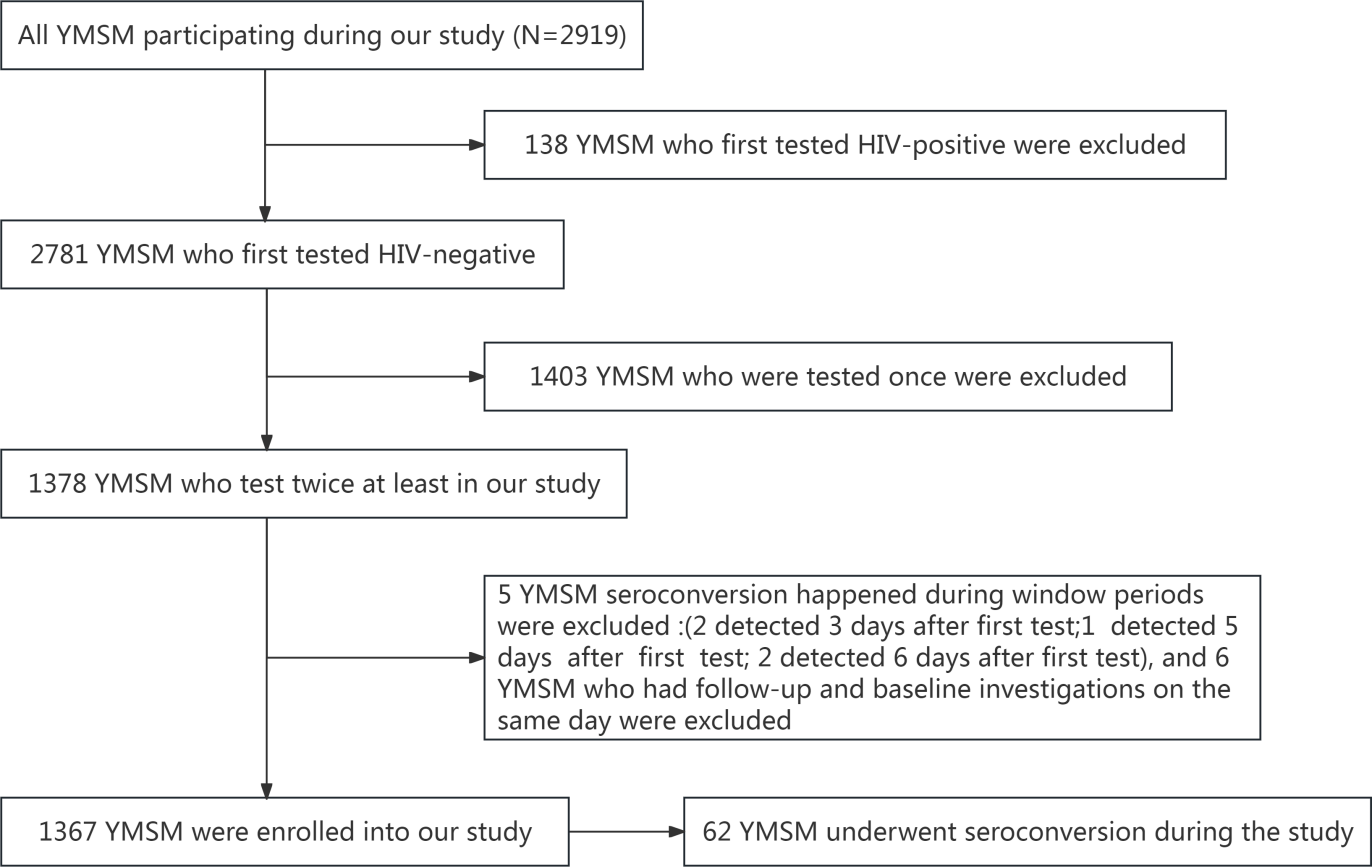
Retrospective cohort study screening process among YMSM in Tianjin, China,2017‐2022. YMSM: young men who have sex with men.

A total of 2919 YMSM completed the baseline survey in this study, of which 138 YMSM yielded positive results for HIV and were excluded from the initial investigation, with 2781 HIV-negative YMSM remaining after the first test. An additional 1403 YMSM who completed only the baseline survey were further excluded from the cohort. A total of 1378 YMSM then participated in two or more surveys during the study. Among these, 6 YMSM who underwent follow-up and baseline investigations on the same day were excluded. Additionally, 5 YMSM were excluded due to positive seroconversion within the window period (7d) after the baseline investigation. Lastly, a total of 1367 HIV-negative YMSM aged 16‐24 years, each with at least two HIV test records were identified and included in our cohort. Among the 1367 HIV-negative YMSM who were enrolled in our retrospective cohort study, 62 YMSM were newly infected with HIV during the study period (Figure 1). All participants in the cohort were followed up and tested simultaneously.

A total of 1403 YMSM were lost to follow-up in our study. Compared to those retained in the cohort YMSM, lost to follow-up YMSM had a higher proportion of out-of-school individuals, a higher frequency of anal intercourse in the last week, lower condom use during their last anal sex experience, and were less likely to use psychoactive substances (Table 1).

**Table 1.** Comparisons of characteristics between the cohort YMSM^a^ and the lost to follow-up YMSM in Tianjin, China, 2017‐2022.

Variables	The cohort YMSM (N=1367), n (%)	The lost to follow-up YMSM (n=1403), n (%)	*P* value
Education level			.67
High school or below	266 (19.5)	282 (20.1)	
College and above	1101 (80.5)	1121 (79.9)	
Duration of residence in Tianjin (years)			.10
<2	304 (22.2)	349 (24.9)	
≧2	1063 (77.8)	1054 (75.1)	
Occupation			.03
Out-of-school YMSM	742 (54.3)	818 (58.3)	
Students YMSM	625 (45.7)	585 (41.7)	
Sexual orientation			.57
Homosexual	1283 (93.9)	1324 (94.4)	
Bisexual/heterosexual	84 (6.1)	79 (5.6)	
Primary venue to find sexual partners			.37
Online	1193 (87.3)	1208 (86.1)	
Offline	174 (12.7)	195 (13.9)	
Number of anal intercourse last week			.02
<2	1127 (82.4)	1109 (79)	
≧2	240 (17.6)	294 (21)	
Used condoms for anal sex last time			.002
No	451 (33)	541 (38.6)	
Yes	916 (67)	862 (61.4)	
Psychoactive substance use			.001
No	699 (51.1)	802 (57.2)	
Yes	668 (48.9)	601 (42.8)	

a YMSM: young men who have sex with men.

### Demographic Characteristics

The majority (1101/1367, 80.5%) of participants completed college or university education. Most participants (n=1063, 77.8%) lived in Tianjin for over two years. A total of 45.7% (n=625) participants were students. The cohort participants’ education level and duration of residence in Tianjin were statistical significant between different HIV seroconversion statuses (*P*<.05), whereas occupation and number of follow-up visits did not significantly differ by different statuses of HIV seroconversion (*P*>.05) (Table 2).

**Table 2.** Demographic characteristics of the cohort YMSM^a^ in Tianjin,China,2017‐2022.

Variables	Participants (N=1367), n (%)	HIV seroconversion		*P* value
		Yes, n (%)	No, n (%)	
Education level				.02
High school or below	266 (19.5)	19 (30.6)	247 (18.9)	
College and above	1101 (80.5)	43 (69.4)	1058 (81.1)	
Duration of residence in Tianjin (years)				.03
<2	304 (22.2)	7 (11.3)	297 (22.8)	
≧2	1063 (77.8)	55 (88.7)	1008 (77.2)	
Occupation				.26
Out-of-school YMSM	742 (54.3)	38 (61.3)	704 (53.9)	
Student YMSM	625 (45.7)	24 (38.7)	601 (46.1)	
Times of follow-up in the study				.47
<3	875 (64)	37 (59.7)	838 (64.2)	
≥3	492 (36)	25 (40.3)	467 (35.8)	

a YMSM: young men who have sex with men.

### Sexual Behaviors, Syphilis Infection, and Psychoactive Substance Use

Among participants, 93.9% (1283/1367) identified themselves as homosexual, and 87.3% (n=1193) mainly found sexual partners online. In the past 6 months, 97.4% (n=1331) had anal intercourse, 17.6% (n=240) had more than two instances of anal intercourse in the last week, and 33.0%(n=451) did not use condoms during their last anal sex experience. Most (n=999, 73.1%) participants had their first homosexual intercourse between the ages of 16‐19 years. Additionally, 7.3% (n=100) were male sex workers and 7.8% (n=107) participants were infected with syphilis. A total of 48.9% (n=668) used psychoactive substances, 25.2% (n=345) were smokers, and 41.6% (n=569) consumed alcohol (Table 3). The rate of psychoactive drug use was higher among smokers (209/345, 60.6%) than nonsmokers (459/1022, 44.9%*; P*<.001). Similarly, the rate of psychoactive drug use was more common among alcohol drinkers (310/569, 54.5%) than nondrinkers at (358/798, 44.9%; *P*<.001).

**Table 3.** Sexual behaviors, syphilis infection, and psychoactive substances use among the cohort YMSM^a^ in Tianjin, China, 2017‐2022.

Variables	Participants (N=1367), n (%)	HIV seroconversion		*P* value
		Yes, n (%)	No, n (%)	
Sexual orientation				.48
Homosexual	1283 (93.9)	60 (96.8)	1223 (93.7)	
Bisexual/heterosexual	84 (6.1)	2 (3.2)	82 (6.3)	
Primary venue to find sexual partners				.02
Online	1193 (87.3)	48 (77.4)	1145 (87.7)	
Offline	174 (12.7)	14 (22.6)	160 (12.3)	
Number of anal intercourse last week				.32
<2	1127 (82.4)	54 (87.1)	1073 (82.2)	
≧2	240 (17.6)	8 (12.9)	232 (17.8)	
Use a condom for anal sex last time				.50
No	451 (33.0)	18 (29)	433 (33.2)	
Yes	916 (67.0)	44 (71)	872 (66.8)	
Age of first homosexual intercourse (years)				.02
<16	70 (5.1)	8 (12.9)	62 (4.8)	
16‐19	999 (73.1)	42 (67.7)	957 (73.3)	
>19	298 (21.8)	12 (19.4)	286 (21.9)	
Syphilis infection				.006
No	1260 (92.2)	51 (82.3)	1209 (92.6)	
Yes	107 (7.8)	11 (17.7)	96 (7.4)	
Male sex worker				.13
No	1267 (92.7)	61 (98.4)	1206 (92.4)	
Yes	100 (7.3)	1 (1.6)	99 (7.6)	
Psychoactive substance use				.001
No	699 (51.1)	19 (30.6)	680 (52.1)	
Yes	668 (48.9)	43 (69.4)	625 (47.9)	
Smoking				.69
No	1022 (74.8)	45 (72.6)	977 (74.9)	
Yes	345 (25.2)	17 (27.4)	328 (25.1)	
Alcohol consumption				.17
No	798 (58.4)	31 (50)	767 (58.8)	
Yes	569 (41.6)	31 (50)	538 (41.2)	
Know about PrEP^b^^c^				.03
No	513 (63.3)	26 (81.3)	487 (62.6)	
Yes	297 (36.7)	6 (18.7)	291 (37.4)	

a YMSM: young men who have sex with other men.

b There is missing data.

c PrEP: pre-exposure prophylaxis.

The primary venue for finding sexual partners, age of first homosexual intercourse, syphilis infection, and psychoactive substance use were statistically significant between different HIV seroconversion statuses (all *Ps*<.05). However, the number of instances of anal intercourse in the last week, condom use during last anal sex, male sex worker status, smoking status, and alcohol consumption status were not significantly associated with HIV seroconversion (all *Ps*>.05) (Table 3).

A total of 95.8% (n=599) student MSM considered themselves homosexual men, which was higher than the proportion among out-of-school YMSM; additionally, 93.0%(n=581) of student YMSM mainly found sexual partners online, which was higher than among out-of-school YMSM. Student YMSM also had lower rates of using condoms for anal sex, syphilis infection, psychoactive substance use, smoking, and alcohol consumption compared to out-of-school YMSM (Table 4).

**Table 4.** Comparisons of characteristics between the out-of-school YMSM^a^ and the student YMSM among the YMSM cohort in Tianjin, China, 2017‐2022.

Variables	Out-of-school YMSM, n (%)	Students YMSM, n (%)	*P* value
Sexual orientation			.005
Homosexual	684 (92.2)	599 (95.8)	
Bisexual/heterosexual	58 (7.8)	26 (4.2)	
Primary venue to find sexual partners			<.001
Online	612 (82.5)	581 (93.0)	
Offline	130 (17.5)	44 (7.0)	
Frequency of anal intercourse last week			<.001
<2	575 (77.5)	552 (88.3)	
≧2	167 (22.5)	73 (11.7)	
Use of a condom for anal sex during last sexual encounter			<.001
No	189 (25.5)	262 (41.9)	
Yes	553 (74.5)	363 (58.1)	
Age at first homosexual intercourse (years)			<.001
<16	42 (5.6)	28 (4.5)	
16‐19	497 (67.0)	502 (80.3)	
>19	203 (27.4)	95 (15.2)	
Syphilis infection			<.001
No	657 (88.5)	603 (96.5)	
Yes	85 (11.5)	22 (3.5)	
Psychoactive substance use			<.001
No	321 (43.3)	378 (60.5)	
Yes	421 (56.7)	247 (39.5)	
Smoking			<.001
No	485 (65.4)	537 (85.9)	
Yes	257 (34.6)	88 (14.1)	
Alcohol consumption			<.001
No	383 (51.6)	415 (66.4)	
Yes	359 (48.4)	210 (33.6)	

a YMSM: young men who have sex with men.

### HIV Incidence and the Associated Factors

**Figure 2.**
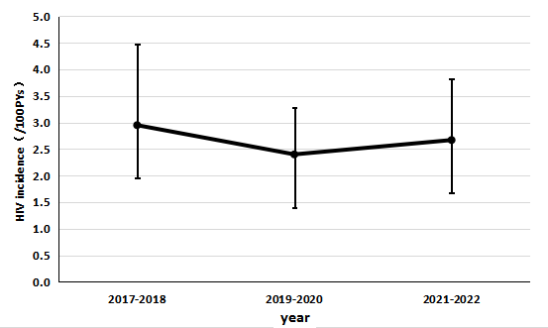
Trends in HIV incidence and 95% CI among YMSM in Tianjin, China, 2017‐2022. PY: person-year. YMSM: young men who have sex with men.

Follow-up encounters ranged from 1 to 20, with the follow-up intervals ranging from 0.4 to 60.3 months with a median value of 16.5 (IQR 8.3‐31.5) months among participants. The total person-time observed was 2384.2 PYs. The overall HIV incidence was 2.6 per 100 PYs (95% CI 2.0‐3.2). To reduce the impact of random errors on the incidence rate and provide a more comprehensive perspective on observing incidence trends, we used a 2-year incidence rate. The segmented HIV incidence rates were 3.0 (95% CI 1.5‐4.5), 2.4 (95% CI 1.5‐3.3), and 2.7 (95% CI 1.5‐3.9) per 100 PYs in 2017‐2018, 2019‐2020, and 2021‐2022, respectively (Figure 2).

Table 5 shows that YMSM who were followed-up at least three times in the study had an incidence of 2.0 per 100 PYs. and were at a lower risk of HIV infection compared to those followed-up fewer than three times during the study (Adjusted hazard ratio [AHR] 0.553, 95% CI 0.325-0.941). The members who found sexual partners offline had an HIV incidence of 4.7 per 100 PYs. These individuals were at more than twice the risk of HIV infection compared to individuals who find sexual partners online (AHR 2.207, 95% CI 1.198-4.066). People infected with syphilis had an HIV incidence of 5.6 per 100 PYs and had a 2.234-fold higher risk of HIV infection compared to those without infected syphilis (AHR 2.234, 95% CI 1.137-4.391). The YMSM who used psychoactive substances had an HIV incidence of 3.7 per 100 PYs and a 2.467-fold greater risk of HIV infection compared to those who did not use psychoactive substances (AHR 2.467, 95% CI 1.408-4.321).

**Table 5.** Factors associated with HIV incidence identified using a proportional hazards regression model among YMSM^a^ in Tianjin, China, 2017‐2022.

Variables	Incidence (per 100 PYs)^b^	Univariate analysis		Multivariate analysis	
		HR^c^ value (95% CI)	*P* value	HR value (95% CI)	*P* value
Education level			.08		.08
High school or below	3.7	1.000		1.000	
College and above	2.3	0.616 (0.359-1.057)		0.593 (0.328-1.073)	
Duration of residence in Tianjin (years)			.07		.13
<2	1.4	1.000		1.000	
≧2	2.9	2.061 (0.938-4.527)		1.887 (0.837-4.252)	
Occupation			.63		.53
Nonstudents	2.7	1.000		1.000	
Students	2.4	0.882 (0.528-1.472)		1.201 (0.678-2.126)	
Times of follow-up in the study			.11		.03
<3	3.2	1.000		1.000	
≧3	2.0	0.658 (0.392-1.104)		0.553 (0.325-0.941)	
Sexual orientation			.31		.65
Homosexual	2.7	1.000		1.000	
Bisexual/heterosexual	1.3	0.484 (0.118-1.978)		0.716 (0.170-3.010)	
Primary venue to find sexual partners			.02		.01
Online	2.3	1.000		1.000	
Offline	4.7	2.054 (1.132-3.725)		2.207 (1.198-4.066)	
Number of anal intercourse last week			.48		.55
<2	2.7	1.000		1.000	
≧2	2.1	0.764 (0.364-1.606)		0.791 (0.369-1.695)	
Use a condom for anal sex last time			.65		.88
No	2.9	1.000		1.000	
Yes	2.5	0.881 (0.508-1.530)		0.956 (0.542-1.687)	
Age of first homosexual intercourse (years)					
<16	6.2	1.000	—^d^	1.000	—^d^
16‐19	2.4	0.383 (0.180-0.815)	.01	0.490 (0.226-1.059)	.07
>19	2.3	0.376 (0.153-0.919)	.03	0.547 (0.217-1.380)	.20
Syphilis Infection			.007		.02
No	2.3	1.000		1.000	
Yes	5.6	2.440 (1.272-4.683)		2.234 (1.137-4.391)	
Male sex worker			.12		.13
No	2.8	1.000		1.000	
Yes	0.8	0.207 (0.029-1.496)		0.209 (0.027-1.614)	
Psychoactive substance use			.002		.002
No	1.6	1.000		1.000	
Yes	3.7	2.369 (1.380-4.065)		2.467 (1.408-4.321)	

a YMSM: young men who have sex with men.

b PY: person-year.

c HR: hazard ratio.

d not applicable.

## Discussion

### Principal Findings

Our study is the first long-term cohort study to assess the incidence of HIV and its associated factors among YMSM in Tianjin. The HIV incidence identified in this study was similar to the incidence among MSM under 24 years old (2.63/100PYs) in Shanghai, published in 2019 [17]. This study found that the incidence of HIV among YMSM in Tianjin showed a stable trend from 2017 to 2022 and was maintained at an elevated level. This steady trend in HIV incidence suggests that risk factors for HIV infection continue to exist; additionally, as a group whose capacity for self-regulation has not fully matured [18], YMSM are more likely to engage in risky behaviors [19]. In our study, approximately 80% of the cohort members had their first homosexual encounter before the age of 20. Individuals who had a sexual debut at an earlier age are more likely to be cognitively immature and involved in high-risk sexual behaviors. Earlier sexual debut is associated with an increased HIV transmission risk [20]. Furthermore, a previous study identified YMSM as potentially more vulnerable to HIV infection than older MSM, reinforcing that YMSM in Tianjin are still at high risk of HIV infection [21].

Our study found a decreased risk of HIV incidence among YMSM who found sexual partners online. In contrast, for depth analysis, among those who found sexual partners offline, the primary venue (135/174, 77.6%) was gay bathhouses, where YMSM are more likely to engage in risky sexual behaviors [22]. Additionally, a study demonstrated that MSM who find partners online have more sexual encounters and multiple partners; however, this behavior was not associated with a greater risk of HIV infection [23]. This may be due to increased distrust and suspicion of partners met online.

As reported in a previous study [24], syphilis infection is a known risk factor associated with HIV incidence, as both HIV and syphilis share the same transmission route [24]. Therefore, we should conduct routine screening and treatment for syphilis infection among YMSM as a measure to prevent HIV infection [25].

Consistent with our findings, previous studies have shown that YMSM who participate in follow-up visits more frequently have a lower HIV incidence [26]. This could be attributed to these individuals being more concerned about HIV infection, which could reduce their sexual risk behaviors, such as decreasing the likelihood of condomless anal sex with a man whose HIV status was unknown. Although data on knowledge of HIV pre-exposure prophylaxis (PrEP) was partially missing in our study, YMSM who experienced HIV seroconversion had a lower awareness rate of HIV PrEP than those who did not. Therefore, we should take effective prevention measures such as PrEP, in addition to routine interventions, to decrease new HIV infections among YMSM.

The use of psychoactive substances was identified as a risk factor for HIV incidence in our study. Additionally, 48.9% of YMSM reported using psychoactive substances, which is higher than that among all MSM in the same region [27]. Psychoactive substances are mostly sold online, and 90% of YMSM in our study mainly found sexual partners online, a higher proportion than among the general MSM population [27]. Furthermore, with the development of network technology and improved internet access, more young people seek health services, particularly HIV-related services, through the internet [28]. Previous studies revealed that MSM who use gay social networking apps reported a higher number of sexual partners, suggesting that we should explore effective intervention strategies using the internet [29,30].

Another finding in our study was that YMSM who smoked or consumed alcohol had a higher prevalence of psychoactive substance use, which is a risk factor for HIV infection. This suggests that strengthening health education initiatives for reducing smoking and alcohol consumption among YMSM may help lower the incidence of HIV.

YMSM in Tianjin remain a vulnerable subpopulation for HIV infection and should be given priority for HIV intervention programs. Ongoing HIV control efforts among young students in schools in Tianjin, such as lectures on HIV, large-scale publicity and education, knowledge contests on HIV prevention, and debate competitions. Given the ongoing HIV epidemic among young students in Tianjin, we designed posters for student YMSM and displayed them in all universities, which yielded a good effect. However, performing effective work is challenging for the out-of-school YMSM due to the absence of a fixed setting, such as a school for intervention. In addition, our study found that, compared to the student YMSM, out-of-school YMSM were more inclined to engage in high-risk behaviors and had a higher proportion of bisexual or heterosexual orientation. Consequently, out-of-school YMSM are more likely to serve as a bridge population for HIV transmission from high-risk MSM to the general population. Therefore, additional prevention options and efforts should be made available to this group.

There are several limitations to this study. First, it was impossible to follow up on the HIV infection status of the individuals who did not enter the cohort; we aim to conduct the follow-up study in the future. Second, as we conducted our study through an NGO, the target population who did not seek HIV testing through the NGO were excluded, potentially leading to selection bias. Finally, given the sensitive nature of the topics, such as sexual behavior, reporting bias may exist.

### Conclusions

HIV incidence among YMSM in Tianjin remains high and has not changed over time. Syphilis infection and the use of psychoactive substances continue to be risk factors associated with HIV incidence. Additionally, risky behaviors such as the use of psychoactive substances are widely present among YMSM; therefore, tailored interventions for this population should be strengthened. Our study supplements the data and evidence for research on new HIV infections among YMSM, both in and out-of-school in China’s megacities and has significant practical implications.

## Abbreviations

AHR: adjusted hazard ratio
MSM: men who have sex with men
NGO: nongovernmental organization
PrEP: pre-exposure prophylaxis
PY: person-years
YMSM: young men who have sex with men
